# Ethnic bias amongst medical students in Aotearoa/New Zealand: Findings from the *Bias and Decision Making in Medicine* (BDMM) study

**DOI:** 10.1371/journal.pone.0201168

**Published:** 2018-08-10

**Authors:** Donna Cormack, Ricci Harris, James Stanley, Cameron Lacey, Rhys Jones, Elana Curtis

**Affiliations:** 1 Te Kupenga Hauora Māori, Faculty of Medical and Health Sciences, University of Auckland, Auckland, New Zealand; 2 Dean’s Department, University of Otago Wellington, Wellington, New Zealand; 3 Māori/Indigenous Health Institute (MIHI), University of Otago Christchurch, Christchurch, New Zealand; Public Library of Science, UNITED KINGDOM

## Abstract

Although health provider racial/ethnic bias has the potential to influence health outcomes and inequities, research within health education and training contexts remains limited. This paper reports findings from an anonymous web-based study examining racial/ethnic bias amongst final year medical students in Aotearoa/New Zealand. Data from 302 students (34% of all eligible final year medical students) were collected in two waves in 2014 and 2015 as part of the Bias and Decision Making in Medicine (BDMM) study. Two chronic disease vignettes, two implicit bias measures, and measures of explicit bias were used to assess racial/ethnic bias towards New Zealand European and Māori (indigenous) peoples. Medical students demonstrated implicit pro-New Zealand European racial/ethnic bias on average, and bias towards viewing New Zealand European patients as more compliant relative to Māori. Explicit pro-New Zealand European racial/ethnic bias was less evident, but apparent for measures of ethnic preference, relative warmth, and beliefs about the compliance and competence of Māori patients relative to New Zealand European patients. In addition, racial/ethnic bias appeared to be associated with some measures of medical student beliefs about individual patients by ethnicity when responding to a mental health vignette. Patterning of racial/ethnic bias by student characteristics was not consistent, with the exception of some associations between student ethnicity, socioeconomic background, and racial/ethnic bias. This is the first study of its kind with a health professional population in Aotearoa/New Zealand, representing an important contribution to further understanding and addressing current health inequities between Māori and New Zealand European populations.

## Introduction

Systematic, disquieting health inequities between indigenous and non-indigenous populations are evident in Aotearoa/New Zealand and many countries internationally [1,2]. Despite this, there has been relatively limited research focused on the role of medical education and workforce development in improving indigenous health and addressing racial/ethnic health inequities. However, growing recognition of the potential for provider bias to influence healthcare interactions and service delivery in ways that maintain or widen racial/ethnic health inequities [3,4] has been accompanied by an increase in studies interrogating racial/ethnic bias amongst health providers [5–10].

### Conceptual approach to racial/ethnic bias

Bias involves “… generally negative feelings and evaluations of individuals because of their group membership (prejudice), overgeneralized beliefs about the characteristics of group members (stereotypes), and inequitable treatment (discrimination)” (p. 201, [4]). Drawing on the work of key scholars [4,8,11–13], racial/ethnic bias in this study refers to beliefs, attitudes, feelings and behaviours about and towards Māori (indigenous peoples of Aotearoa/New Zealand) and NZ European (the numerically dominant population) people. Critically, racial/ethnic bias at an individual level is understood as an expression of the broader phenomenon of racism [4] within which ‘racial’/‘ethnic’ groups are produced and have social meaning. Manifestations of racial/ethnic bias can be explicit and direct, or subtle and less conscious [4,14,15]. Studies of health provider racial/ethnic bias often involve assessment of both “conscious and intentional” bias (p. 201, [4]), or *explicit bias*, as well as *implicit bias* that involves “unintentional activation, often outside personal awareness” (p.102, [14]). While the expression of overt racial bias may be influenced by social norms around acceptability, implicit bias is less susceptible to influences of social desirability [8,15,16]. Research has shown that people can hold implicit beliefs and attitudes about racial/ethnic groups that may contrast with the views they consciously express [6,14,17].

### Health provider racial/ethnic bias

Health provider racial/ethnic biases may influence health outcomes and inequities via several pathways [4,7,10]. Provider biases could directly influence clinical decision-making processes and outcomes, including management and treatment decisions (e.g., [3,18]) to disadvantage some groups and advantage others [7]. Provider racial/ethnic bias may also impact healthcare interactions through influencing the quality of communication and experience for patients, with possible flow-on effects, such as likelihood of following recommendations and satisfaction with care (e.g., [18,19]).

There is a small but growing body of quantitative research assessing health provider racial/ethnic bias and its relationship to clinical decision-making, almost exclusively US-based [4–10]. These studies have identified relatively consistent pro-White bias relative to African American and Latino/a populations, as well as bias towards Native American populations (e.g., [5–10,20,21]). In studies that have assessed both explicit and implicit racial bias among healthcare providers, explicit bias tends to be reported at lower levels (e.g., [22–26]), a finding consistent with literature about racial bias more broadly (e.g., [6,15]). Implicit and explicit racial/ethnic bias have been shown to not be strongly correlated with each other (e.g., [25–27]), aligning with literature that suggests the constructs are capturing different processes as well as reflecting social desirability effects [11,15].

The association between provider bias and differential clinician diagnosis, management and treatment decision-making is variable [5,7,9]. However, evidence for the association of provider bias on clinician-patient interaction, such as communication or satisfaction with care, appears more consistent (e.g., [7,10,28]). Only a few studies have been with medical student populations [24,29–31] or examined health provider racial/ethnic bias towards indigenous peoples [20,21].

### Aim and hypotheses

To our knowledge, there have been no quantitative studies of medical student (or other health professional student) implicit and explicit racial/ethnic bias in Aotearoa/New Zealand, despite evidence of racialised beliefs and stereotypes about Māori in general (e.g., [32] and Māori patients in particular (e.g., [33]). It is likely, however, that medical students will be exposed to narratives about Māori, in both their medical education and broader social interactions, that include racially-biased discourses.

The *Bias and Decision Making in Medicine* (BDMM) study aimed to examine implicit and explicit racial/ethnic bias in relation to Māori and NZ European people amongst medical students. In line with the extant research [5–10] and local evidence on the existence of generalised beliefs about Māori patients [33], we hypothesised that medical student racial/ethnic bias would exist in Aotearoa/New Zealand, and that it may differ by student characteristics. We also hypothesised that explicit racial/ethnic bias would be lower than implicit bias, and that implicit and explicit racial/ethnic bias would not be highly correlated at the group level, reflecting both social desirability bias and the different processes involved [15,16,27]. In addition, this paper explores associations between both implicit and explicit racial/ethnic bias and responses to bias items in two hypothetical clinical scenarios about individual NZ European or Māori patients.

## Methods

The BDMM study is a sub-project of the *Educating for Equity* international collaborative project. It was approved by the University of Auckland Human Ethics Committee (Reference 011693) and ratified by the University of Otago Ethics Committee. Study development and testing is detailed elsewhere [34].

### Participants

The medical degree in Aotearoa/New Zealand is a six-year undergraduate degree. All final year (i.e. sixth year) medical students at both Aotearoa/New Zealand medical schools were invited to participate. Two waves of data collection were undertaken: 4–16 November 2014 (n = 438 eligible students completing their final year); and, 27 January–8 February 2015 (n = 450 eligible students early in their final year).

Eligible participants were sent an initial email invitation and general email reminders via administrative staff. Emails contained brief study information, a link to the web-based study, and a common password to gain access. Overall, 302 final year medical students participated in the study (34% of eligible participants). Study participants were similar in gender, age, and ethnicity to the total eligible sample (S1 Table). Detail on participation and retention across study modules is appended (S1 Fig).

### Procedures

The study comprised four modules: 1) basic demographics; 2) two clinical vignettes; 3) two Implicit Association Tests (IATs); and, 4) explicit bias measures and additional demographics. The study entry page contained the participant information sheet and consent form. This page explained that the study was exploring bias among medical students and potential impacts on clinical decision-making, but did not specify racial/ethnic bias, in order to reduce any bias in responding to clinical vignettes by randomised patient ethnicity. The focus on bias towards NZ European or Māori became apparent in the third and fourth modules (following the vignettes). The modules were ordered to reduce social desirability bias in line with comparable studies among clinicians [6, 23,27,35].

The main web-based study was deployed via Qualtrics™. The IATs were developed with Project Implicit and hosted on Project Implicit servers, with participants automatically linked out and back in to the main questionnaire. Debriefing information was provided at the end of the study, followed by an option to be redirected to a separate website to receive a $20 voucher and/or entry into a draw for $500 of vouchers.

### Measures

The measures used in this study are outlined below. Further detail on the measures is included in a separate methods paper [34], including information on the identification, development and piloting of study tools.

#### Implicit racial/ethnic bias

The IAT measures the speed with which respondents associate specific stimuli (e.g., images of people) with particular attributes (e.g., positive and negative words) [36]. Two localised IATs were developed to compare Māori with NZ European ethnic groups [34].

The ‘ethnicity preference IAT’ (Preference IAT), adapted from the *race preference IAT* [37], contained prototypical Māori and NZ European images (3 matched female pairs, 3 matched male pairs), with the labels “Māori” and “NZ European”. Image stimuli for the IAT were developed specifically for this study. Full details of the construction and validation processes have been previously described [34]. Briefly, headshot photos for sixteen volunteers were matched between apparent ethnicities based on several factors (e.g. hair length, weight, apparent age). The final set of images were selected following construct testing for prototypicality of the apparent ethnicity of the photographed individual [34]. The attributes (positive and negative words) were from the race preference IAT (joy, love, peace, wonderful, pleasure, glorious, laughter, happy, agony, terrible, horrible, nasty, evil, awful, failure, hurt) [37].

The ‘ethnicity and compliant patient IAT’ (Compliance IAT) was adapted from the *race and compliant patient IAT* [27], and used the same images as the Preference IAT, with “compliant patient” and “reluctant patient” labels. Word stimuli included six words relating to “compliant patient” (willing, cooperative, compliant, reliable, adherent, helpful) and six to “reluctant patient” (reluctant, averse, hesitant, apathetic, resistant, slack) [27,34].

From these IATs, D scores were calculated using a standard process [36] to derive a score between -2 and +2. Negative scores indicate preference/higher compliance for Māori relative to NZ European images, and positive scores indicate the opposite. D scores are reported using previously published cutoffs to categorise bias [36]. IAT results from two participants were excluded according to data quality/processing rules around speed and precision of IAT responses [36,38].

#### Explicit racial/ethnic bias

Explicit ethnic preference was measured by asking participants to rate their preference for NZ Europeans relative to Māori on a 7-point scale, where ‘1’ represented strong preference for NZ Europeans, ‘4’ neutral, and ‘7’ strong preference for Māori (adapted from [23,36]). Responses were reverse scored in analyses for consistency across measures.

Respondents rated their feelings of warmth toward Māori and NZ European ethnic groups separately, from ‘1’ = “least warm” to ‘7’ = “most warm” (adapted from [39]). Within-participant differences in warmth ratings were calculated, with positive values indicating greater warmth for Māori compared to NZ European.

Generalised beliefs about patient groups were assessed using six questions about perceived compliance and competence of Māori and NZ European patients: *“In general*, *how [competent/intelligent/confident] do you think [NZ European/Māori] patients are*?*”* (adapted from [40]); and, “*In general*, *how [compliant/reliable/motivated] do you think [NZ European/Māori] patients are*?*”* (adapted from [40,41]). Questions were asked separately for each ethnic group, with responses on a 7-point scale from ‘Not at all’ to ‘Extremely’. The mean responses from individual items were computed to calculate Total Compliance and Total Competence scores. Mean paired differences between responses for Māori and NZ European were calculated for each individual item, and for the two Total scores.

#### Vignettes

Clinical decision making was assessed with a cardiovascular disease (CVD) and a mental health vignette. Ethnicity of the patient (Māori or NZ European) and vignette order were randomly assigned. Ethnicity was marked by reference to the patient as either Māori or NZ European and use of a Māori or English language surname (Mr Wiremu/Williams or Mr Tipene/Stephens), with all other elements of the vignette held identical. The vignettes included questions on clinical decision-making (not reported here), and on beliefs or expectations of the patient [34]. The beliefs and expectations questions comprised three items on the CVD vignette (likelihood of patient refusing treatment, likelihood of patient understanding medical advice, level of comfort working with patient) and four on the mental health vignette (reliability of patient’s information, likelihood of forming a good relationship with the clinician, likelihood of patient taking anti-depressant medication, likelihood of patient attending recommended specialist appointment). Response options for each item were on a 5-point scale.

#### Demographic variables

Initial demographic variables included: gender (male, female, other); age (in years); and self-identified ethnicity using the standard New Zealand Census ethnicity question [42]. Responses were categorised for analysis into aggregate groupings (Māori, Pacific, Asian, Other, European), with multiple ethnicity responses prioritised into one analytical category according to established procedures [43]. Other variables included: socioeconomic background growing up (low, lower-middle, middle, upper-middle, high) (adapted from [23]); nativity (born in Aotearoa/New Zealand, yes or no) and year of arrival if born outside New Zealand [44]. Social-desirability was measured using the Rand-5 Social Desirability Response Set (SDRS) [45].

### Analysis methods

Data were analysed using R 3.1.2 (R Institute, Vienna, Austria). Means and standard deviations, frequencies and percentages describe the demographic profile of participants. Frequencies and means (where relevant) were calculated for the implicit and explicit bias measures, alongside mean paired differences for the warmth, competence, and compliance items.

Multivariable linear regression analysis was used to examine the relationship between explicit and implicit bias. For the Preference IAT outcome, associations are reported for ethnic preference and warmth difference (as two separate analyses.) For the Compliance IAT outcome, results are reported according to the total compliance score difference and the total competence score difference (as two separate analyses).

Associations between demographic characteristics and implicit/explicit bias measures were examined using linear regression. Each bias measure was treated as the outcome in a separate analysis: adjusted associations for each demographic characteristic (age, gender, SES, ethnicity, and nativity) from fully adjusted models are reported alongside unadjusted mean responses for each group. The coefficients from these models represent mean differences in the outcome measure relative to a reference category for each predictor/factor.

We also examined the association between implicit/explicit bias measures and vignette item responses using linear regression. As participants were randomised to either the NZ European or Māori patient vignette, we examined the association between bias and vignette responses as slopes within each patient ethnicity group, and used interaction terms to examine the differential association by patient ethnicity (as differences in slopes).

## Results

Participant characteristics are detailed in Table 1. Respondents’ median age was 24 years, and 47% were male, although gender proportions differed by study wave. Most participants identified with a European ethnic group, reported a middle or upper-middle socioeconomic background, and were born in Aotearoa/New Zealand.

**Table 1:** Characteristics of study participants, by wave and combined.

Characteristic	Wave 1	Wave 2	Combined
	n (%)	n (%)	n (%)
	Total n = 120	Total n = 182	Total n = 302
*Prioritised ethnicity*			
NZ European	69 (57)	105 (58)	174 (58)
Māori	8 (7)	10 (5)	18 (6)
Pacific	5 (4)	4 (2)	9 (3)
Asian	33 (28)	61 (34)	94 (31)
Other	5 (4)	2 (1)	7 (2)
*Age*^*a*^			
Median (IQR)	24 (23–25)	23 (23–24)	24 (23–25)
*Missing*	*3 (3)*	*0 (0)*	*3 (1)*
*Gender*			
Male	68 (57)	74 (41)	142 (47)
Female	52 (43)	108 (59)	160 (53)
*Self-reported SES*			
Low	4 (3)	4 (2)	8 (3)
Lower-middle	20 (17)	26 (14)	46 (15)
Middle	34 (28)	50 (27)	84 (28)
Upper-middle	30 (25)	58 (32)	88 (29)
High	8 (7)	7 (4)	15 (5)
*Not reported*	*24 (20)*	*37 (20)*	*61 (20)*
*Born in New Zealand*			
Yes	55 (46)	96 (53)	151 (50)
No	41 (34)	49 (27)	90 (30)
*Not reported*	*24 (20)*	*37 (20)*	*61 (20)*
*SDRS*^*b*^			
0	55 (46)	68 (37)	123 (41)
1	21 (18)	48 (26)	69 (23)
2	14 (12)	19 (10)	33 (11)
3	6 (5)	9 (5)	15 (5)
4	0 (0)	0 (0)	0 (0)
5	0 (0)	2 (1)	2 (1)
*Not reported*	*24 (20)*	*36 (20)*	*60 (20)*

Data beneath the dotted line was collected in the final module. Missing data reflects drop-out prior to this.

^a^ Top response category was “Aged 30+”, treated as 30 for calculation of median and IQR.

^b^ Higher scores indicate higher social desirability [34].

### Implicit racial/ethnic bias

**Fig 1:**
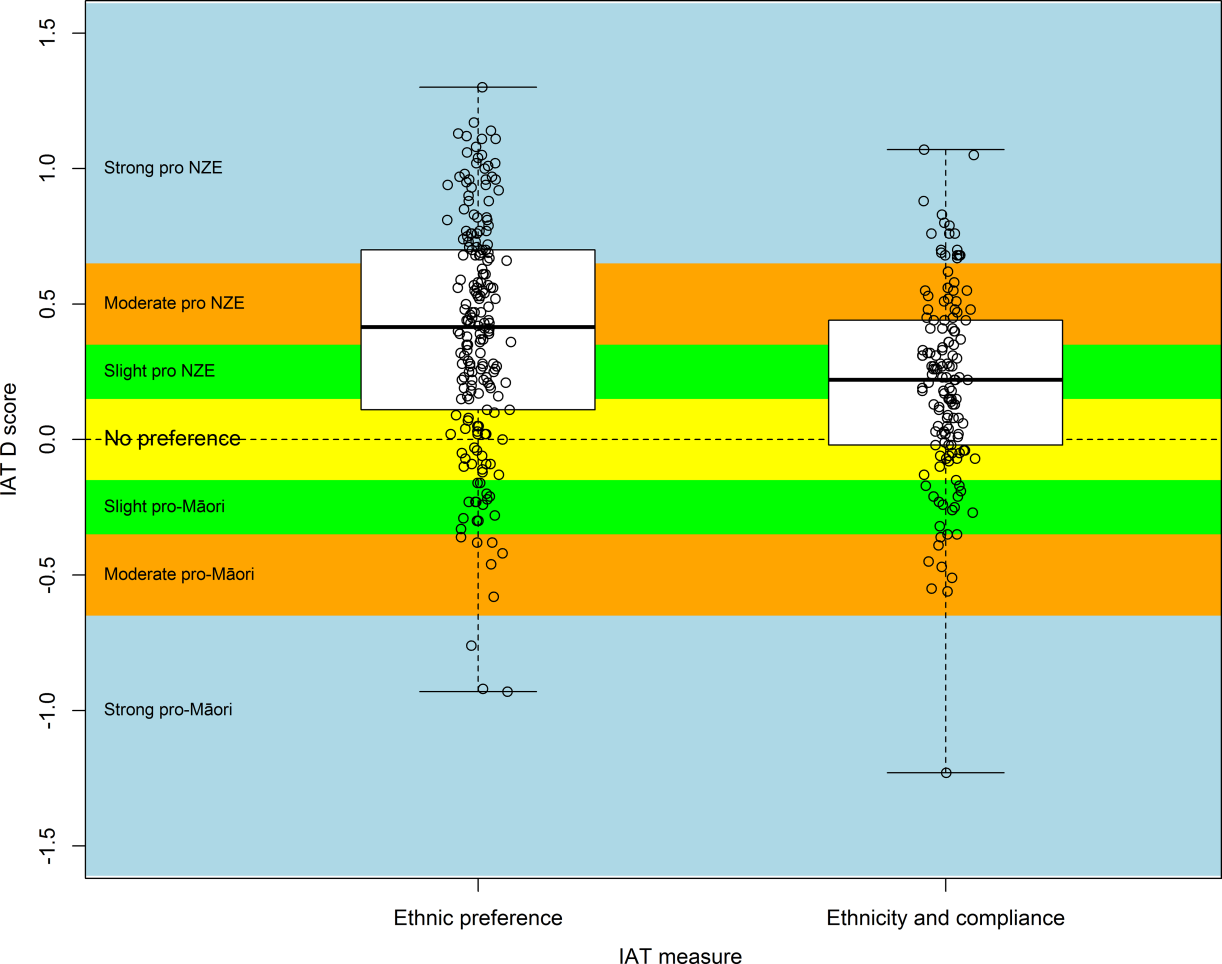
Distribution of D scores for ethnic preference (n = 198) and ethnicity and compliant patient (n = 144) IATs. Legend: D scores range from -2 to +2. Negative scores indicate implicit preference/implicit higher compliance for Māori, positive scores indicate implicit preference/implicit higher compliance for NZ Europeans.

Responses for the Preference IAT (n = 198) indicated implicit preference for NZ Europeans relative to Māori on average across participants, with a mean D = 0.39 (95% CI 0.33, 0.45; one-sample t-test (df = 197) p < 0.001), in the “moderate preference” range (Fig 1). For the Compliance IAT (n = 144), participants indicated “slightly” higher implicit association of NZ European patients with compliance attributes relative to Māori patients, with a mean D = 0.20 (95% CI 0.14, 0.26; one-sample t-test (df = 197) p < 0.001) (Fig 1).

### Explicit racial/ethnic bias

**Fig 2:**
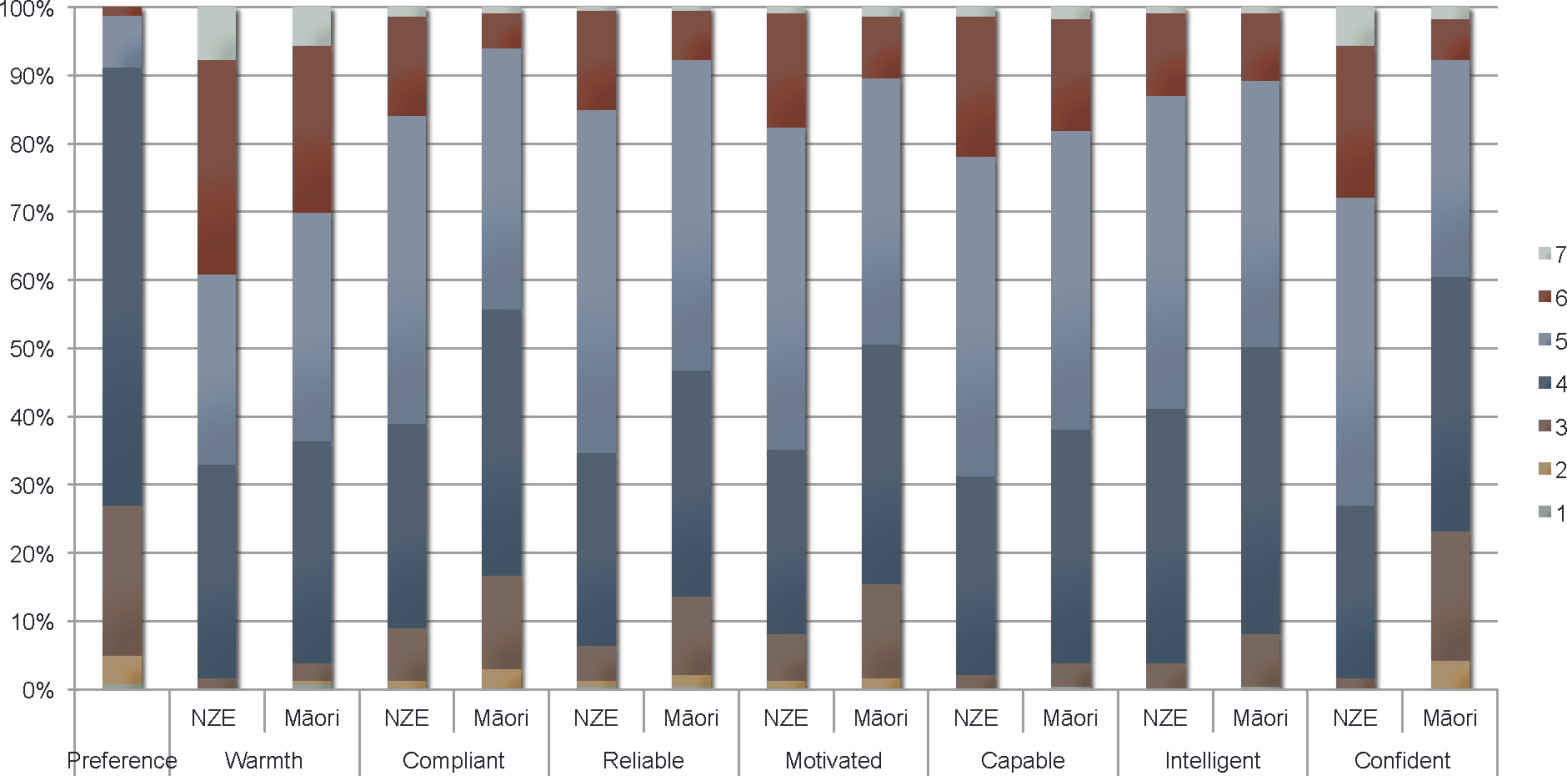
Distribution of responses to explicit bias questions. Legend: ^a^ Ethnic preference response options: 1 = I strongly prefer Māori to NZ Europeans, 2 = I moderately prefer Māori to NZ Europeans, 3 = I slightly prefer Māori to NZ Europeans, 4 = I like NZ Europeans and Māori equally, 5 = I slightly prefer NZ Europeans to Māori, 6 = I moderately prefer NZ Europeans to Māori, 7 = I strongly prefer NZ Europeans to Māori. ^c^ Compliance and competence response options: ‘1’ = “Not at all” to ‘7’ = “Extremely”.

Most participants (65%) indicated they liked Māori and NZ Europeans equally (Fig 2). However, approximately one-quarter indicated some preference for NZ Europeans (relative to Māori), while 9% reported some preference for Māori. The mean ethnic preference score was 4.22 (95% CI 4.12, 4.31), indicating a slight average preference for NZ Europeans relative to Māori (Table 2).

**Table 2:** Responses to explicit bias measures.

Item	Mean	Mean paired difference
	(95% CI)	(95% CI)
***Ethnic preference***^***a***^*** ***	4.2 (4.1, 4.3)	-
***Warmth***^***b***^		
*Warmth towards NZE*	5.1 (5.0, 5.3)	0.19 (0.06, 0.32)
*Warmth towards Māori*	4.9 (4.8, 5.1)	
***Compliance***^***c***^		
*Compliant NZE*	4.7 (4.6, 4.0)	0.36 (0.26, 0.47)
*Compliant Māori*	4.3 (4.2, 4.3)	
*Reliable NZE*	4.7 (4.6, 4.8)	0.27 (0.18, 0.37)
*Reliable Māori*	4.5 (4.3, 4.6)	
*Motivated NZE*	4.7 (4.6, 4.9)	0.30 (0.19, 0.42)
*Motivated Māori*	4.4 (4.3, 4.6)	
***Competence***^***c***^		
*Capable NZE*	4.9 (4.8, 5.0)	0.13 (0.06, 0.19)
*Capable Māori*	4.8 (4.7, 4.9)	
*Intelligent NZE*	4.7 (4.6, 4.8)	0.16 (0.09, 0.24)
*Intelligent Māori*	4.5 (4.4, 4.6)	
*Confident NZE*	5.1 (4.9, 5.2)	0.83 (0.68, 0.98)
*Confident Māori*	4.2 (4.1, 4.4)	

^a^ Ethnic preference response options: 1 = I strongly prefer Māori to NZ Europeans, 2 = I moderately prefer Māori to NZ Europeans, 3 = I slightly prefer Māori to NZ Europeans, 4 = I like NZ Europeans and Māori equally, 5 = I slightly prefer NZ Europeans to Māori, 6 = I moderately prefer NZ Europeans to Māori, 7 = I strongly prefer NZ Europeans to Māori

^b^ Warmth response options: ‘1’ = “least warm” to ‘7’ = “most warm”

^c^ Compliance and competence response options: ‘1’ = “Not at all” to ‘7’ = “Extremely”.

Overall, the mean warmth score towards NZ Europeans was higher than towards Māori (Table 2). Among people who answered both questions, the mean warmth rating for NZ Europeans was 0.19 points higher than for Māori (95% CI 0.06, 0.32; paired sample t-test (df = 232), p = 0.0053), although most people rated both groups equally (65%; Fig 2).

Participants gave lower ratings on compliance and competence items for Māori than for NZ European patient groups (Table 2), as assessed by mean paired differences. The mean composite Total Compliance score was higher for NZ European patients (4.7, 95% CI 4.6, 4.8), compared to Māori (4.4, 95% CI 4.3, 4.5), giving a mean paired difference of 0.31 (95% CI 0.23, 0.40; paired sample t-test (df = 232), p<0.001). The mean composite Total Competence score was also higher for NZ European patients (4.9, 95% CI 4.8, 5.0), than for Māori (4.5, 95% CI 4.4, 4.6; mean paired difference = 0.37, 95% CI 0.30, 0.44; paired sample t-test (df = 232), p<0.001).

### Association between implicit and explicit bias

Implicit ethnic preference was weakly associated with explicit ethnic preference (n = 193; mean difference in IAT score per one-unit difference in preference for NZ European = 0.10, 95% CI 0.02, 0.19; df = 191, p = 0.019) and with the warmth paired difference (n = 193, mean difference in IAT score per one-unit difference in higher warmth rating for NZ European = 0.08, 95% CI = 0.01, 0.15; df = 142 p = 0.025).

The Compliance IAT was weakly correlated with explicit compliance (n = 144; mean difference in IAT score per one-unit higher total compliance score for NZ European = 0.12, 95% CI 0.02, 0.22; p = 0.025), but the association with explicit competence was not significant (mean difference in IAT score per one-unit higher total compliance score for NZ European = 0.05, 95% CI = -0.08, 0.17; p = 0.467).

### Associations with medical student characteristics

There were no significant differences according to student characteristics for most adjusted analyses for implicit (Table 3) or explicit (Table 4) racial/ethnic bias. Implicit preference for NZ Europeans appeared to increase with higher socioeconomic background, but a similar relationship was not apparent for Compliance IAT scores. Although the lowest socioeconomic grouping had the lowest explicit bias in unadjusted results, they were not significantly different from the reference group after adjustment (Table 4).

**Table 3:** Association of implicit racial/ethnic bias measures with participant characteristics.

		Preference IAT (n = 198)			Compliance IAT (n = 144)	
	n	Unadjusted mean^a^	Adjusted difference	n	Unadjusted mean^a^	Adjusted difference
*Factor*		(95% CI)	(95% CI)		(95% CI)	(95% CI)
*Age*^*b*^						
21–23	108	0.42 (0.35, 0.49)	Reference	83	0.24 (0.19, 0.29)	Reference
24–26	71	0.37 (0.29, 0.45)	-0.05 (-0.18, 0.08)	48	0.12 (0.05, 0.19)	-0.11 (-0.24, 0.02)
27+	17	0.34 (0.22, 0.45)	0.05 (-0.19, 0.28)	12	0.29 (0.15, 0.42)	0.15 (-0.08, 0.38)
*Gender*						
M	88	0.46 (0.38, 0.53)	Reference	59	0.18 (0.11, 0.24)	Reference
F	110	0.34 (0.28, 0.41)	-0.11 (-0.23, 0.02)	85	0.22 (0.16, 0.27)	0.08 (-0.05, 0.20)
*SES*^*c*^						
Low	6	0.26 (0.00, 0.52)	-0.18 (-0.55, 0.19)	3*	-	-
Lower-middle	34	0.34 (0.22, 0.46)	0.04 (-0.14, 0.22)	22	0.13 (0.04, 0.22)	-0.05 (-0.24, 0.13)
Middle	71	0.37 (0.27, 0.48)	Reference	56	0.17 (0.10, 0.23)	Reference
Upper-middle	72	0.41 (0.33, 0.49)	0.08 (-0.07, 0.23)	55	0.26 (0.17, 0.34)	0.11 (-0.04, 0.25)
High	10	0.67 (0.47, 0.88)	0.33 (0.05, 0.61)	8	0.19 (-0.05, 0.44)	0.07 (-0.20, 0.35)
*Prioritised ethnicity*						
NZ European	119	0.36 (0.30, 0.43)	Reference	90	0.19 (0.13, 0.24)	Reference
Māori	12	0.04 (-0.18, 0.26)	-0.32 (-0.59, -0.06)	8	0.11 (-0.05, 0.26)	-0.03 (-0.30, 0.24)
Pacific	5	0.40 (0.14, 0.65)	0.09 (-0.32, 0.50)	2*	-	-
Asian	60	0.52 (0.44, 0.60)	0.10 (-0.05, 0.26)	43	0.25 (0.19, 0.32)	0.10 (-0.06, 0.25)
Other	2*	-	-	1*	-	-
*Nativity*^*d*^						
Born in NZ	121	0.33 (0.26, 0.39)	Reference	91	0.18 (0.13, 0.23)	Reference
Born overseas	72	0.50 (0.41, 0.60)	0.12 (-0.03, 0.26)	53	0.24 (0.16, 0.32)	0.05 (-0.09, 0.20)

^a^ D scores range from -2 to +2. Negative scores indicate implicit preference/implicit higher compliance for Māori, positive scores indicate implicit preference/implicit higher compliance for NZ Europeans.

^b^ n = 2 participants missing age for Preference IAT; 1 missing for Compliance IAT; older ages are grouped.

^c^ n = 5 participants missing SES for Preference IAT.

^d^ n = 5 participants missing Nativity for Preference IAT.

*Included in data analysis; results not presented to preserve privacy.

**Table 4:** Association of explicit racial/ethnic bias measures with participant characteristics.

		Ethnic Preference^a^			Warmth difference^b^		Compliance mean paired difference^b^		Competence mean paired difference^b^	
	n	Unadjusted mean	Adjusted difference	n	Unadjusted mean	Adjusted difference	Unadjusted mean	Adjusted difference	Unadjusted mean	Adjusted difference
*Factor*		(95% CI)	(95% CI)		(95% CI)	(95% CI)	(95% CI)	(95% CI)	(95% CI)	(95% CI)
*Age*^*c*^										
21–23	125	4.26 (4.13, 4.38)	Reference	125	0.12 (-0.05, 0.29)	Reference	0.25 (0.16, 0.35)	Reference	0.41 (0.32, 0.50)	Reference
24–26	85	4.24 (4.11, 4.36)	-0.09 (-0.29, 0.12)	85	0.33 (0.15, 0.51)	0.16 (-0.13, 0.46)	0.37 (0.24, 0.50)	0.08 (-0.10, 0.26)	0.33 (0.23, 0.44)	-0.12 (-0.28, 0.04)
27+	20	3.95 (3.66, 4.24)	-0.26 (-0.64, 0.11)	20	-0.05 (-0.41, 0.31)	-0.12 (-0.66, 0.41)	0.37 (0.14, 0.59)	0.23 (-0.10, 0.55)	0.37 (0.21, 0.52)	0.01 (-0.27, 0.30)
*Gender*										
M	108	4.23 (4.10, 4.37)	Reference	108	0.25 (0.05, 0.45)	Reference	0.33 (0.22, 0.44)	Reference	0.37 (0.28, 0.47)	Reference
F	125	4.21 (4.11, 4.31)	-0.08 (-0.27, 0.12)	125	0.14 (0.01, 0.27)	-0.13 (-0.42, 0.15)	0.30 (0.21, 0.40)	0.06 (-0.11, 0.23)	0.38 (0.29, 0.46)	0.03 (-0.12, 0.18)
*SES*										
Low	8	3.88 (3.43, 4.32)	-0.16 (-0.72, 0.40)	8	-0.12 (-0.91, 0.66)	-0.14 (-0.94, 0.66)	0.08 (-0.12, 0.29)	-0.16 (-0.64, 0.33)	0.12 (-0.05, 0.30)	-0.35 (-0.78, 0.08)
Lower-middle	43	4.05 (3.87, 4.22)	-0.13 (-0.41, 0.15)	43	0.00 (-0.18, 0.18)	-0.14 (-0.54, 0.26)	0.30 (0.12, 0.49)	0.00 (-0.25, 0.24)	0.29 (0.18, 0.41)	-0.16 (-0.37, 0.06)
Middle	84	4.19 (4.01, 4.37)	Reference	84	0.10 (-0.13, 0.32)	Reference	0.33 (0.19, 0.47)	Reference	0.48 (0.33, 0.62)	Reference
Upper-middle	85	4.35 (4.21, 4.50)	0.08 (-0.15, 0.32)	85	0.40 (0.16, 0.64)	0.28 (-0.06, 0.61)	0.31 (0.18, 0.44)	0.11 (-0.09, 0.31)	0.29 (0.19, 0.39)	-0.13 (-0.31, 0.05)
High	13	4.31 (3.93, 4.69)	0.04 (-0.40, 0.47)	13	0.23 (-0.14, 0.60)	0.06 (-0.56, 0.68)	0.38 (-0.03, 0.80)	0.20 (-0.18, 0.58)	0.67 (0.36, 0.97)	0.27 (-0.07, 0.60)
*Ethnicity*^*d*^										
NZ European	139	4.28 (4.19, 4.37)	Reference	139	0.24 (0.11, 0.38)	Reference	0.20 (0.12, 0.28)	Reference	0.33 (0.26, 0.40)	Reference
Māori	14	3.71 (3.38, 4.05)	-0.37 (-0.79, 0.05)	14	-0.21 (-0.54, 0.11)	-0.16 (-0.77, 0.44)	0.52 (0.07, 0.97)	0.40 (0.04, 0.77)	0.45 (0.15, 0.75)	0.13 (-0.20, 0.45)
Pacific	6	3.33 (2.80, 3.87)	-0.97 (-1.61, -0.32)	6	-0.33 (-0.67, 0.00)	-0.44 (-1.36, 0.49)	0.11 (-0.22, 0.44)	0.13 (-0.43, 0.69)	0.56 (0.20, 0.91)	0.36 (-0.13, 0.86)
Asian	72	4.28 (4.10, 4.45)	-0.07 (-0.31, 0.18)	72	0.21 (-0.06, 0.47)	-0.03 (-0.38, 0.32)	0.50 (0.36, 0.65)	0.29 (0.07, 0.50)	0.45 (0.31, 0.59)	0.06 (-0.13, 0.25)
*Nativity*										
Born in NZ	145	4.17 (4.06, 4.27)	Reference	145	0.14 (-0.02, 0.31)	Reference	0.22 (0.13, 0.31)	Reference	0.32 (0.24, 0.39)	Reference
Born Overseas	88	4.31 (4.13, 4.48)	0.1 (-0.14, 0.33)	88	0.26 (0.06, 0.46)	0.07 (-0.26, 0.40)	0.46 (0.31, 0.62)	0.18 (-0.02, 0.38)	0.47 (0.34, 0.60)	0.15 (-0.02, 0.33)
*SDRS*										
0	116	4.30 (4.16, 4.45)	Reference	123	0.28 (0.08, 0.48)	Reference	0.37 (0.26, 0.48)	Reference	0.38 (0.27, 0.49)	Reference
1	68	4.16 (4.00, 4.32)	-0.13 (-0.35, 0.09)	69	0.13 (-0.08, 0.34)	-0.10 (-0.42, 0.22)	0.23 (0.07, 0.39)	-0.15 (-0.35, 0.04)	0.34 (0.24, 0.45)	-0.07 (-0.24, 0.10)
2	32	4.00 (3.83, 4.17)	-0.27 (-0.57, 0.03)	33	-0.16 (-0.46, 0.15)	-0.39 (-0.81, 0.03)	0.32 (0.10, 0.55)	-0.10 (-0.36, 0.15)	0.47 (0.28, 0.66)	0.07 (-0.15, 0.30)
3	15	4.33 (3.97, 4.70)	0.08 (-0.32, 0.49)	15	0.47 (0.00, 0.93)	0.17 (-0.41, 0.75)	0.27 (-0.03, 0.56)	-0.06 (-0.41, 0.29)	0.36 (0.16, 0.55)	0.04 (-0.27, 0.35)
4	0	-	-	0						
5	2	0 (—)	0 (—)	2	0 (—)	0 (—)	0 (—)	-0.39 (-1.33, 0.54)	0 (—)	-0.60 (-1.43, 0.22)

^a^ Ethnic preference reverse scored. Scores above 4 (neutral) indicate preference for NZE relative to Māori, and scores below 4 indicate preference for Māori relative to NZE.

^b^ Mean paired difference.

^c^ Age missing n = 3 responses for Ethnic Preference.

^d^ Ethnicity is prioritised, ‘Other’ ethnic grouping not included because of small numbers.

Numbers less than 5 have been censored.

On average, Māori participants demonstrated no implicit racial/ethnic bias for either IAT in unadjusted results. Following adjustment, Māori participants had significantly lower implicit scores for the Preference IAT, compared with responses from NZ European participants (Table 3). Māori and Pacific students had lower unadjusted explicit ethnic preference scores (i.e., tended to have a preference for Māori) and lower mean warmth difference than the other ethnic groupings. Pacific students’ mean adjusted explicit preference scores were significantly lower than NZ European students’ (-0.97, 95% CI -1.61, -0.32). Māori and Asian students tended to have the largest difference in mean paired compliance scores (i.e. rating compliance for NZ European higher than for Māori) in both unadjusted and adjusted estimates (Table 4).

### Associations with vignette responses

There was little difference in mean scores for vignette bias items between those randomly assigned the Māori or NZ European patient, with the exception of higher comfort ratings for the Māori patient (mean -0.24, 95% CI -0.07, -0.01; df = 278, p = 0.045) (S2 Table).

Further analysis examined whether implicit or explicit bias was associated with differential responding to the vignette bias items. In the mental health vignette, higher explicit preference for NZ Europeans was associated with lower rating of patient information as reliable for those assigned the Māori, but not the NZ European, patient (significant difference in slopes = 0.27, 95% CI 0.02, 0.51). Higher explicit preference for NZ Europeans was also associated with rating NZ European patients as more likely to form a therapeutic relationship with their GP and more likely to take their antidepressant medication as prescribed, with significant differences in slope compared to Māori patients (Table 5).

**Table 5:** Associations between racial/ethnic bias measures and response to vignette bias questions by vignette patient ethnicity.

Vignette question bias measures (number in analysis by patient ethnicity)	Patient Described as:		Difference (NZE-Māori) in slope (95% CI)
	NZ European slope (95% CI)	Māori slope (95% CI)	
**Cardiovascular disease vignette**			
*Likelihood [patient] will ultimately refuse thrombolysis after further discussion*^*a*^			
-Preference IAT (NZE = 95, Māori = 103)	-0.14 (-0.56, 0.28)	0.02 (-0.36, 0.40)	-0.16 (-0.73, 0.40)
-Compliance IAT (NZE = 75, Māori = 69)	-0.16 (-0.75, 0.42)	-0.40 (-0.94, 0.15)	0.23 (-0.57, 1.03)
-explicit ethnic preference (NZE = 123, Māori = 118)	0.11 (-0.11, 0.33)	-0.05 (-0.28, 0.17)	0.16 (-0.15, 0.48)
-explicit ethnic warmth paired difference (NZE = 123, Māori = 118)	0.02 (-0.14, 0.17)	-0.07 (-0.23, 0.09)	0.08 (-0.14, 0.31)
-explicit compliance paired difference (NZE = 123, Māori = 118)	0.18 (-0.09, 0.45)	0.08 (-0.23, 0.39)	0.11 (-0.30, 0.51)
*Likelihood [patient] understands medical advice regarding thrombolysis*^*b*^			
-Preference IAT (NZE = 95, Māori = 103)	0.25 (-0.26, 0.75)	-0.14 (-0.60, 0.32)	0.38 (-0.30, 1.07)
-Compliance IAT (NZE = 75, Māori = 69)	0.12 (-0.58, 0.81)	-0.20 (-0.85, 0.45)	0.32 (-0.63, 1.27)
-explicit ethnic preference (NZE = 123, Māori = 118)	0.18 (-0.07, 0.44)	-0.10 (-0.35, 0.16)	0.28 (-0.08, 0.64)
-explicit ethnic warmth paired difference (NZE = 123, Māori = 118)	0.07 (-0.11, 0.26)	0.00 (-0.19, 0.19)	0.08 (-0.19, 0.34)
-explicit competence paired difference (NZE = 123, Māori = 118)	0.17 (-0.02, 0.36)	-0.05 (-0.31, 0.21)	0.22 (-0.10, 0.54)
*Level of comfort working with [patient]*^*c*^			
-Preference IAT (NZE = 95, Māori = 103)	-0.32 (-0.79, 0.14)	-0.66 (-1.08, -0.23)	0.33 (-0.30, 0.96)
-Compliance IAT (NZE = 75, Māori = 69)	-0.40 (-1.07, 0.27)	-0.55 (-1.18, 0.08)	0.15 (-0.77, 1.07)
-explicit ethnic preference (NZE = 123, Māori = 118)	0.03 (-0.21, 0.27)	-0.16 (-0.41, 0.08)	0.19 (-0.15, 0.53)
-explicit ethnic warmth paired difference (NZE = 123, Māori = 118)	-0.02 (-0.19, 0.15)	-0.09 (-0.26, 0.09)	0.06 (-0.18, 0.31)
**Mental health vignette**			
*Reliability of information provided by this patient*^*d*^			
-Preference IAT (NZE = 105, Māori = 193)	-0.18 (-0.48, 0.12)	-0.24 (-0.56, 0.08)	0.06 (-0.38, 0.50)
-Compliance IAT (NZE = 75, Māori = 69)	0.08 (-0.31, 0.47)	-0.34 (-0.74, 0.07)	0.42 (-0.14, 0.98)
-explicit ethnic preference (NZE = 125, Māori = 116)	0.09 (-0.08, 0.27)	-0.17 (-0.34, 0.00)	0.27 (0.02, 0.51)
-explicit ethnic warmth paired difference (NZE = 125, Māori = 116)	0.05 (-0.09, 0.19)	0.09 (-0.03, 0.20)	-0.03 (-0.21, 0.15)
-explicit compliance paired difference (NZE = 125, Māori = 116)	-0.23 (-0.40, -0.06)	-0.20 (-0.45, 0.04)	-0.03 (-0.32, 0.27)
*Likelihood [patient] will form a good therapeutic alliance with his GP*^*e*^			
-Preference IAT (NZE = 105, Māori = 93)	0.19 (-0.15, 0.53)	-0.17 (-0.53, 0.20)	0.35 (-0.14, 0.85)
-Compliance IAT (NZE = 75, Māori = 69)	0.20 (-0.28, 0.68)	-0.42 (-0.92, 0.08)	0.62 (-0.08, 1.31)
-explicit ethnic preference (NZE = 125 Māori = 116)	0.25 (0.06, 0.44)	-0.05 (-0.23, 0.13)	0.29 (0.03, 0.56)
-explicit ethnic warmth paired difference (NZE = 125, Māori = 116)	-0.13 (-0.28, 0.02)	-0.03 (-0.15, 0.09)	-0.10 (-0.30, 0.09)
-explicit compliance paired difference (NZE = 125, Māori = 116)	-0.06 (-0.25, 0.12)	0.06 (-0.20, 0.33)	-0.13 (-0.45, 0.20)
*Likelihood [patient] will take anti-depressant medication as prescribed*^*e*^			
-Preference IAT (NZE = 105, Māori = 93)	0.16 (-0.19, 0.50)	-0.37 (-0.73, 0.00)	0.52 (0.02, 1.02)
-Compliance IAT (NZE = 75, Māori = 69)	0.00 (-0.48, 0.49)	-0.26 (-0.76, 0.24)	0.26 (-0.43, 0.96)
-explicit ethnic preference (NZE = 125, Māori = 116)	0.20 (0.01, 0.39)	-0.21 (-0.39, -0.03)	0.41 (0.15, 0.67)
-explicit ethnic warmth paired difference (NZE = 125, Māori = 116)	-0.10 (-0.25, 0.05)	0.08 (-0.04, 0.21)	-0.18 (-0.38, 0.01)
-explicit compliance paired difference (NZE = 125, Māori = 116)	-0.06 (-0.25, 0.12)	-0.09 (-0.36, 0.18)	0.03 (-0.3, 0.35)
*Likelihood [patient] will attend appointment for assessment by specialist mental health services*^*e*^			
-Preference IAT (NZE = 105, Māori = 93)	0.07 (-0.29, 0.42)	-0.13 (-0.51, 0.24)	0.20 (-0.32, 0.71)
-Compliance IAT (NZE = 75, Māori = 69)	0.53 (0.05, 1.01)	-0.11 (-0.61, 0.39)	0.64 (-0.06, 1.33)
-explicit ethnic preference (NZE = 125, Māori = 116)	0.14 (-0.06, 0.34)	-0.08 (-0.26, 0.11)	0.22 (-0.06, 0.49)
-explicit ethnic warmth paired difference (NZE = 125, Māori = 116)	-0.10 (-0.26, 0.06)	0.01 (-0.12, 0.13)	-0.11 (-0.31, 0.09)
-explicit compliance paired difference (NZE = 125, Māori = 116)	-0.20 (-0.38, -0.01)	-0.31 (-0.58, -0.04)	0.11 (-0.22, 0.44)

^a^Response options reverse scored (1 = very likely (<80%), 2 = somewhat likely (60–80%), 3 = as likely as not (41–59%), 4 = somewhat unlikely (20–40%), 5 = very unlikely (>80%))

^b^Response options (1 = very unlikely (<20%), 2 = somewhat unlikely (20–40%), 3 = as likely as not (41–59%), 4 = somewhat likely (60–80%), 5 = very likely (>80%))

^c^Response options (1 = very uncomfortable, 2 = somewhat uncomfortable, 3 = neutral, 4 = somewhat comfortable, 5 = very comfortable)

^d^Response options (1 = very unreliable, 2 = somewhat unreliable, 3 = as reliable as not, 4 = somewhat reliable, 5 = very reliable)

^e^Response options (1 = very unlikely, 2 = somewhat unlikely, 3 = as likely as not, 4 = somewhat likely, 5 = very likely)

There were fewer differential associations between implicit racial/ethnic bias and vignette responding by patient ethnicity. Higher implicit preference for NZ Europeans (compared to Māori) was associated with rating Māori patients as less likely to take their antidepressant medication, with a significant difference in the slopes (Table 5). Implicit association of compliance with NZ European patients was associated with rating the NZ European patient as more likely to attend their specialist mental health appointment, but not for the Māori patient (Table 5).

## Discussion

This is the first study to report frequency and patterning of implicit and explicit racial/ethnic bias among medical students in Aotearoa/New Zealand, adding to the small pool of similar studies internationally involving medical students [24,30,31] or assessing health provider bias towards indigenous peoples [20,21]. We found evidence of pro-NZ European (dominant ethnic group) bias among this medical student cohort, with most respondents indicating some level of implicit preference for NZ Europeans, and also for implicit association of NZ Europeans with positive compliance attributes relative to Māori. This aligns with international studies that find implicit bias favouring the dominant racial/ethnic group among medical students [24,30], physicians (e.g., [5,19,22]) and other health providers [8]. Mean D scores in this study are similar to those reported among first year medical students [24], medical students in their first, third and fourth years [30], and physician groups in the United States for the race/ethnic preference IAT (e.g., (19,22]) and the race/ethnicity and compliant patient IAT (e.g., [19,35]). Mean D scores for the ethnicity and compliant patient IAT were lower than for the ethnic preference IAT in our study. Studies with physicians in the United States found similar or higher mean D scores for the race and compliant patient IAT relative to the race preference IAT [19,35]. Mean D scores for the compliance IAT in our study were similar to those among physicians in the United States [19,35]. Potentially, generalised assumptions about racial/ethnic groups may be more salient for medical students in Aotearoa/New Zealand than those more specific to healthcare settings, while attitudes about patient compliance may increase over time as time in clinical environments increases.

In line with our hypothesis, explicit racial/ethnic bias was less apparent than implicit bias, with medical students tending to rate ethnic groups equally on explicit measures. However, on average, medical students did demonstrate some explicit racial/ethnic bias for NZ Europeans relative to Māori, for all explicit measures. As with implicit bias, explicit ethnic preference findings were similar to those reported for first year medical students in the United States regarding explicit racial bias towards Black individuals relative to White individuals [24].

Medical students rated Māori patient groups lower on average for all measures of compliance and competence, with the biggest difference in paired ratings for the ‘confidence’ item. Responding to this measure may be influenced by medical student beliefs about the responsiveness and cultural safety of the health system that could impact on Māori patient confidence in healthcare interactions. There also appeared to be patterning of compliance ratings by student ethnicity. The lower ratings for explicit compliance items align with qualitative evidence of generalised beliefs about Māori compliance in terms of health (e.g., [33]). More broadly, these findings may link to the *Stereotype Content* model [14,46] that contends that stereotypes “… express generalised evaluative beliefs that vary according to the degree of *warmth* and *competence* ascribed to members of the target group” (p. 26, [40]). Sibley and colleagues (2011) highlight the influence of the relative social and structural power of ethnic groups as influencing competence appraisals, and beliefs about perceived competition between groups as influencing assessments of warmth. In their study of perceived societal stereotypes about ethnic groups in New Zealand, Sibley et al. (2011) described ‘meta-stereotypes’ with NZ Europeans assessed as high in both competence and warmth relative to other ethnic groupings (Asian, Pacific peoples and Māori), while Māori were seen as being “low-to-moderate” for both warmth and competence. In this sense, our study findings measuring warmth and competence amongst individual participants align with perceptions of the societal stereotypes about NZ Europeans relative to Māori [40].

As hypothesised, implicit and explicit bias were weakly correlated (consistent with similar studies, e.g. [24]), supporting the argument that implicit and explicit measures capture related but different aspects of racial/ethnic bias [11] and the utility of examining both in studies of health provider bias [9].

There appeared to be some relationship between respondent ethnicity and bias in this study, and between respondent socioeconomic status and bias, although these were not entirely consistent across the different racial/ethnic bias measures, potentially due to small numbers in some groups. Student ethnicity has been shown to be associated with racial/ethnic bias in other studies [24,30]. In a study by Haider et al. (2011), Black medical students had mean D scores suggesting no implicit preference in the Race IAT, similar to the finding for Māori in our study that suggested Māori students had no implicit racial/ethnic bias [24]. Blair et al (2013) highlight the need to reflect on individuals who demonstrate no racial/ethnic bias, in terms of the potential to learn from how these individuals maintain low or no racial/ethnic bias in a racialised social context [22].

Students randomly assigned a Māori patient vignette indicated more comfort working with the patient than those assigned a NZ European patient, despite Māori patients being rated lower on average in the warmth ratings. Similar findings have been reported among physicians in the US [47], although the reason for this finding is not clear.

Finally, our study found some associations between implicit or explicit racial/bias measures and vignette bias items (i.e. at the individual patient level) for the mental health, but not the CVD, scenario. This contrasts with the study by Haider et al. (2011) that found no associations between either implicit or explicit race preference and vignette responses. Specifically, explicit ethnic preference for NZ Europeans at a group level was associated with beliefs and stereotypes about individual hypothetical patients in the mental health vignette, in a way that more negatively characterised Māori patients. These results, however, need to be interpreted with regard to the number of comparisons made. Adjustments for multiple comparisons (e.g. Bonferroni correction) would have led to a reduced number of findings being labelled as statistically significant. Whilst unable to be assessed in this study, these beliefs about patients at the group and individual level have the potential to impact on clinical encounters, affecting both provider behavior and patient response to the interaction. Associations have been identified between clinician bias (both implicit race and race and compliance bias) and both communication in patient-clinician interactions and patient ratings of healthcare [19]. Implicit racial/ethnic bias associations have also been reported with measures of communication (e.g., [19,38]), patient-provider interactions (e.g., [28,47]), patient experiences (e.g., [19,48]) and patient outcomes, such as adherence [49].

### Limitations

The response rate was relatively low, and therefore students who participated in the study may not be representative of the final year medical student cohort overall, although they were broadly representative in terms of age, gender, and ethnicity (S1 Table). Low response rates in later medical school years have been shown in other studies [30]. In addition, as bias was only measured at one time point it is unknown whether student racial/ethnic bias increased, decreased or remained static over their medical education. In a US study that included medical students, there was no difference in bias prevalence between early and late year students [30], although longitudinal evidence would be needed to assess changes over time.

It is difficult to assess whether the bias findings for the medical student population are representative of general population bias levels due to a lack of routine monitoring of racial/ethnic bias towards different ethnic groups in Aotearoa/New Zealand. In one US study, implicit racial/ethnic bias was comparable between clinicians and community members [22].

There are some limitations to the vignette measures in that they measure hypothetical scenarios rather than actual healthcare interactions. Response patterns may differ from actual encounters as participants were responding to clinical vignette scenarios under conditions that are likely more relaxed and less time-pressured than actual clinical encounters. There is evidence to support the notion that stereotypes and biases are invoked more in situations of high cognitive load [12]. In addition, there is the potential for the ordering of the modules to influence responding. However, this should be accounted for in the randomisation process.

### Implications

Our finding of pro-NZ European racial/ethnic bias suggests such bias needs to be considered in medical education and, more broadly, in understanding and addressing ethnic health inequities between Māori and NZ European populations in Aotearoa/New Zealand. Although racial/ethnic bias was associated with only some of the bias measures in the hypothetical clinical scenarios, there is the potential for racial/ethnic bias to influence health outcomes both directly, such as through treatment and management decisions, as well as indirectly, including through differential quality of communication in healthcare interactions (e.g., [8,10,22]). In particular, the racial/ethnic bias shown in terms of beliefs about the compliance and competence of Māori patients relative to NZ European patients may be important in relation to chronic conditions, where management is on-going over the long-term, and there is potentially greater discretion in decision-making about treatment and management options.

While intervention is not necessarily simple, it is possible to reduce racial/ethnic bias [12] and mitigate the impacts of racial/ethnic bias on health [10]. Medical education provides opportunities for interventions with medical students to reduce racial/ethnic bias, increase awareness of the potential impacts of bias, and support students to develop strategies to mitigate bias. Exploring racial/ethnic bias among medical students also advances understanding of potential impacts on both students and educators of teaching indigenous health in a racialised society, and may aid efforts to improve learning environments, with flow-on effects for indigenous health teaching initiatives.

## Conclusions

As our understanding of racism as a fundamental health determinant develops, it is important to consider the complex and multi-faceted role that health provider racial/ethnic bias may play in influencing health outcomes and inequities. As racial/ethnic bias amongst individual health providers is a manifestation of a broader context of pervasive exposure to racism and racialised discourses at a societal level, it is therefore not surprising that racial/ethnic bias exists in this cohort of students. This broader context needs to be taken into account in interventions to address racial/ethnic bias among health professionals.

## Supporting information

S1 Fig Participation in study modules, numbers and percentages. (DOCX)

S1 Table Characteristics of study participants, by wave, overall, and compared to total eligible sample. Table notes:
* Top response category was “Aged 30+”, treated as 30 for calculation of median and IQR.
(DOCX)

S2 Table Responses to vignette bias questions by randomised patient ethnicity, means and mean differences. Table notes:
^a^ The ethnicity of the patient in the vignette was randomised. These are two separate groups. Also note that the composition of groups is different for the "Presented with CVD vignette with patient as European" and "Presented with mental health vignette with patient as European" groups.
^b^ Response options reverse scored (1 = very likely (<80%), 2 = somewhat likely (60–80%), 3 = as likely as not (41–59%), 4 = somewhat unlikely (20–40%), 5 = very unlikely (>80%))
^c^ Response options (1 = very unlikely (<20%), 2 = somewhat unlikely (20–40%), 3 = as likely as not (41–59%), 4 = somewhat likely (60–80%), 5 = very likely (>80%))
^d^ Response options (1 = very uncomfortable, 2 = somewhat uncomfortable, 3 = neutral, 4 = somewhat comfortable, 5 = very comfortable)
^e^ Response options (1 = very unreliable, 2 = somewhat unreliable, 3 = as reliable as not, 4 = somewhat reliable, 5 = very reliable)
^f^ Response options (1 = very unlikely, 2 = somewhat unlikely, 3 = as likely as not, 4 = somewhat likely, 5 = very likely.
(DOCX)
