# Supplementary material for: Ethnic bias amongst medical students in Aotearoa/New Zealand: Findings from the Bias and Decision Making in Medicine (BDMM) study
Source: PLoS One. 2018 Aug 10;13(8):e0201168. doi: 10.1371/journal.pone.0201168 (PMC6086411; doi:10.1371/journal.pone.0201168)
Supplement: S1 Fig — (DOCX) [file pone.0201168.s001.docx]

**S1 Fig: Participation in study modules, numbers and percentages**

All final year medical students in both NZ medical schools in 2014 and 2015: n=888

Did not respond to invitation: n=580

Responded but did not initiate questionnaire: n=5

Signed in, but ineligible: n=1

Participated in study: n=302

(34% of all final year medical students)

Participated in demographic module: n=302

Dropped out before Vignette Module: n=15

Participated in Vignette Module: n=287

Dropped out after first vignette: n=7

Dropped out after second vignette: n=31

Still in study prior to IAT module: n=249

Participated in IAT Module: n=203

(Completed Ethnic Preference IAT: n= 198)

(Completed Compliant Patient IAT: n=144)

(Completed both IATs: n=141)

Dropped out after Ethnic Preference IAT: n=7

Dropped out after Ethnicity and Compliant Patient IAT: n=1

Skipped the IAT module: n=41

Removed from IAT due to touch device: n = 5

Removed from IAT responses due to speed/error profile: n = 2

Missing Preference IAT, completed compliance IAT: n=3

Missing compliance IAT, completed preference IAT: n=52

Participated in Explicit Bias Module: n=241

Removed from explicit bias module due to touch device: n=5

Final group for analysis: n=236
