# Supplementary material for: Ethnic bias amongst medical students in Aotearoa/New Zealand: Findings from the Bias and Decision Making in Medicine (BDMM) study
Source: PLoS One. 2018 Aug 10;13(8):e0201168. doi: 10.1371/journal.pone.0201168 (PMC6086411; doi:10.1371/journal.pone.0201168)
Supplement: S1 Table — Table notes: * Top response category was “Aged 30+”, treated as 30 for calculation of median and IQR. (DOCX) [file pone.0201168.s002.docx]

**S1 Table: Characteristics of study participants, by wave, overall, and compared to total eligible sample**

| **Characteristic** | **Wave 1**  **n (%)**  **Total n=120** | **Wave 2**  **n (%)**  **Total n=182** | | **Combined**  **n (%)**  **Total n=302** | | **Total sample**  **(%)**  **Total n=888** |
| --- | --- | --- | --- | --- | --- | --- |
| ***Ethnicity (prioritised)*** | | |  | |  | |
| European | 69 (57) | 105 (58) | | 174 (58) | | (51) |
| Māori | 8 (7) | 10 (5) | | 18 (6) | | (9) |
| Pacific | 5 (4) | 4 (2) | | 9 (3) | | (5) |
| Asian | 33 (28) | 61 (34) | | 94 (31) | | (32) |
| Other | 5 (4) | 2 (1) | | 7 (2) | | (3) |
| ***Age **** |  |  | |  | |  |
| Median (IQR) | 24 (23–25) | 23 (23–24) | | 24 (23–25) | | 24 |
| *Missing* | *3 (3)* | *0 (0)* | | *3 (1)* | |  |
| ***Gender*** |  |  | |  | |  |
| Male | 68 (57) | 74 (41) | | 142 (47) | | (47) |
| Female | 52 (43) | 108 (59) | | 160 (53) | | (53) |

* Top response category was “Aged 30+”, treated as 30 for calculation of median and IQR.
