# Supplementary material for: Ethnic bias amongst medical students in Aotearoa/New Zealand: Findings from the Bias and Decision Making in Medicine (BDMM) study
Source: PLoS One. 2018 Aug 10;13(8):e0201168. doi: 10.1371/journal.pone.0201168 (PMC6086411; doi:10.1371/journal.pone.0201168)
Supplement: S2 Table — Table notes: a The ethnicity of the patient in the vignette was randomised. These are two separate groups. Also note that the composition of groups is different for the "Presented with CVD vignette with patient as European" and "Presented with mental health vignette with patient as European" groups. b Response options reverse scored (1 = very likely (<80%), 2 = somewhat likely (60–80%), 3 = as likely as not (41–59%), 4 = somewhat unlikely (20–40%), 5 = very unlikely (>80%)) c Response options (1 = very unlikely (<20%), 2 = somewhat unlikely (20–40%), 3 = as likely as not (41–59%), 4 = somewhat likely (60–80%), 5 = very likely (>80%)) d Response options (1 = very uncomfortable, 2 = somewhat uncomfortable, 3 = neutral, 4 = somewhat comfortable, 5 = very comfortable) e Response options (1 = very unreliable, 2 = somewhat unreliable, 3 = as reliable as not, 4 = somewhat reliable, 5 = very reliable) f Response options (1 = very unlikely, 2 = somewhat unlikely, 3 = as likely as not, 4 = somewhat likely, 5 = very likely. (DOCX) [file pone.0201168.s003.docx]

**S2 Table: Responses to vignette bias questions by randomised patient ethnicity, means and mean differences**

| **Vignette question** | | **Patient Described as**^a^**:** | | |  |  |
| --- | --- | --- | --- | --- | --- | --- |
|  | | **NZ European (n = 142)**  **mean (95% CI)** | | **Māori (n = 140)**  **mean (95% CI)** | **Mean difference**  **difference (95% CI)** | **p-value** |
| **Cardiovascular disease vignette items** |  | |  | | |  |
| How would you rate the likelihood that Mr [Wiremu/Williams] will ultimately refuse thrombolysis after further discussion?^b^ | | 2.10 (1.96, 2.24) | | 2.19 (2.03, 2.34) | -0.09 (-0.29, 0.12) | 0.399 |
| How would you rate the likelihood that Mr [Wiremu/Williams] understands medical advice regarding thrombolysis?^c^ | | 3.21 (3.04, 3.38) | | 3.21 (3.05, 3.38) | 0.00 (-0.24, 0.23) | 0.980 |
| How comfortable or uncomfortable would you feel working with Mr [Wiremu/Williams]?^d^ | | 3.63 (3.48, 3.79) | | 3.87 (3.70, 4.04) | -0.24 (-0.47, -0.01) | 0.045 |
|  | | **Patient Described as**^a^**:** | | |  |  |
|  | | **NZ European (n = 142)**  **mean (95% CI)** | | **Māori (n = 138)**  **mean (95% CI)** |  |  |
| **Mental health vignette items** | |  | |  |  |  |
| How reliable do you think the information provided by this patient is?^e^ | | 4.09 (3.97, 4.21) | | 4.10 (3.98, 4.22) | -0.01 (-0.18, 0.16) | 0.908 |
| What is the likelihood that Mr [Tipene/Stephens] will form a good therapeutic alliance with his GP?^f^ | | 3.51 (3.39, 3.63) | | 3.46 (3.34, 3.59) | 0.05 (-0.13, 0.22) | 0.600 |
| How would you rate the likelihood that Mr [Tipene/Stephens] will take his anti-depressant medication as prescribed?^f^ | | 3.58 (3.45, 3.71) | | 3.56 (3.44, 3.68) | 0.02 (-0.16, 0.20) | 0.829 |
| How would you rate the likelihood of Mr [Tipene/Stephens] attending his appointment for assessment by specialist mental health services?^f^ | | 3.50 (3.36, 3.63) | | 3.57 (3.44, 3.70) | -0.07 (-0.26, 0.11) | 0.443 |

^a^ The ethnicity of the patient in the vignette was randomised. These are two separate groups. Also note that the composition of groups is different for the "Presented with CVD vignette with patient as European" and "Presented with mental health vignette with patient as European" groups.

^b^ Response options reverse scored (1=very likely (<80%), 2=somewhat likely (60-80%), 3=as likely as not (41-59%), 4=somewhat unlikely (20-40%), 5=very unlikely (>80%))

^c^ Response options (1=very unlikely (<20%), 2=somewhat unlikely (20-40%), 3=as likely as not (41-59%), 4=somewhat likely (60-80%), 5=very likely (>80%))

^d^ Response options (1=very uncomfortable, 2=somewhat uncomfortable, 3=neutral, 4=somewhat comfortable, 5=very comfortable)

^e^ Response options (1=very unreliable, 2=somewhat unreliable, 3= as reliable as not, 4=somewhat reliable, 5=very reliable)

^f^ Response options (1=very unlikely, 2=somewhat unlikely, 3=as likely as not, 4=somewhat likely, 5=very likely
